# Volatile Fatty Acids Production from Microalgae Biomass: Anaerobic Digester Performance and Population Dynamics during Stable Conditions, Starvation, and Process Recovery

**DOI:** 10.3390/molecules24244544

**Published:** 2019-12-12

**Authors:** Jose Antonio Magdalena, Elia Tomás-Pejó, Cristina González-Fernández

**Affiliations:** Biotechnological Processes Unit, IMDEA Energy, 28935 Madrid, Spain; joseantonio.magdalena@imdea.org (J.A.M.); elia.tomas@imdea.org (E.T.-P.)

**Keywords:** anaerobic digestion, disturbance, microalgae, population dynamics, volatile fatty acids

## Abstract

Disturbances in anaerobic digestion (AD) negatively impact the overall reactor performance. These adverse effects have been widely investigated for methane generation. However, AD recently appeared as a potential technology to obtain volatile fatty acids (VFAs) and thus, the impact of process disturbances must be evaluated. In this sense, microbial response towards a starvation period of two weeks was investigated resulting in a conversion of organic matter into VFAs of 0.39 ± 0.03 COD-VFAs/CODin. However, the lack of feeding reduced the yield to 0.30 ± 0.02 COD-VFAs/CODin. Microbial analysis revealed that the starvation period favored the syntrophic acetate-oxidizing bacteria coupled with hydrogenotrophic methanogens. Finally, the system was fed at 9 g COD/Ld resulting in process recovery (0.39 ± 0.04 COD-VFAs/CODin). The different microbiome obtained at the end of the process was proved to be functionally redundant, highlighting the AD robustness for VFAs production.

## 1. Introduction

Replacing products obtained from fossil fuels with others obtained from renewable resources is becoming a worldwide issue. A shift to bio-based chemicals seems crucial to circumvent the negative effects of petrochemicals in the environment and overcome supply limitations. As a matter of fact, the so-called “bioeconomy” aims at the gradual use of renewable feedstocks. Among those marketed fossil fuels derivatives, carboxylates, also known as volatile fatty acids (VFAs), could be produced using an alternative route [1]. Acetic, propionic, (iso) butyric, (iso) valeric, and caproic acid are VFAs that account from two to six carbons. The interest in these compounds as platform molecules lies in their wide chemical industry applications [2].

VFAs are produced during the middle stages (acidogenesis and acetogenesis) of anaerobic digestion (AD). Typically, VFAs are anaerobically oxidized to acetate, which is the main substrate that methanogens use to produce biogas. However, VFAs production from AD requires digestion shortening to avoid the methanogenic step favoring VFAs accumulation. The biochemical process for VFAs production pretends to exploit what should be normally prevented in digesters devoted for biogas production. AD for biogas production is a robust technology applied to a wide range of organic substrates. Several research studies regarding microbial response to disturbances during biogas production were mainly targeted at evaluating this latter AD stage [3,4]. These investigations pointed out to methanogenesis as the most sensitive step due to the slow growth rates and susceptibility to inhibitory substances of methanogens [5,6]. However, the new interest in VFAs makes of utmost importance the study of the bacterial response to disturbances as well as the crucial inhibition of the archaea community to achieve competitive process yields.

With regard to the substrate, the use of microalgae biomass presents potential advantages for the process because of the high protein content exhibited by some strains. During AD, proteins degradation results in the release of ammonium and free ammonia to the medium, which could cause the destabilization of the AD process. As a matter of fact, high concentration of these compounds is toxic for methanogenic archaea, which in turn promotes VFAs accumulation [6].

Common process disturbances studied in the context of biogas production include temperature changes, salinity stress, or feeding alterations [7,8,9]. As feedstock availability can fluctuate along the year, it is important to assess the effects and to propose proper management strategies to overcome this event. Starvation and organic overloading are frequent perturbations in full scale AD, which might affect the microbiome behavior [10]. A microbial ecosystem will be considered resistant when no changes are observed upon a perturbation. Alternatively, if the microbial population is sensitive and does change, it could be resilient and quickly recover to its initial composition. Finally, if the perturbed population is sensitive and displaced by other microorganisms with similar function, the microbial system can be considered functionally redundant. 

In this sense, controlled perturbation experiments can provide useful information in terms of fermenters performance and microbial community dynamics. Suitable VFA yields associated with certain bacterial species can contribute to further proposing recovery strategies and quickly anticipate process failure as well as identifying key organic acid producers. This investigation was designed to cover the gap of knowledge related to the effect that potential perturbations can cause in fermentative processes for VFAs production. With this objective, this investigation evaluated VFA yields and the bacterial and archaea response towards starvation. Population dynamics analysis throughout the different scenarios (stable operation, starvation, feeding re-start, recovery) was assessed to find out the involved microorganisms developing key roles in VFAs production.

## 2. Material and Methods

### 2.1. Inoculum and Substrate Pretreatment 

Temperature and substrate adapted anaerobic sludge was collected from a previous anaerobic reactor set at psychrophilic range temperature (25 °C) and fed with enzymatic pretreated *Chlorella vulgaris*. This acidogenic reactor was previously established for VFAs production. More details regarding characterization of the effluent in the stationary state of this reactor, which was used as anaerobic inoculum in the present study, can be found elsewhere [11]. The substrate *C. vulgaris* was purchased from Allmicroalgae (Lisbon, Portugal) revealing a composition (% *w*/*w* dry weight) of 57.9 proteins, 21.6 carbohydrates, 13.4 lipids and 7.1 ash. Since the goal of this study was to investigate the acidogenic stage, biomass pretreatment was applied to avoid hydrolysis limitation. Commercial enzymatic cocktail “Alcalase 2.5 L” (Novozyme, Denmark) was employed to pretreat the biomass and make available the organic matter to anaerobic microorganisms. The dosage (0.585 UA g^−1^ TS^−1^) and procedure was based on results obtained for *C. vulgaris* [6].

### 2.2. Experimental Set up

Anaerobic fermentation was carried out under semi-continuous feeding mode in 1 L-CSTR. Agitation was performed by magnetic stirring. The operational temperature and hydraulic retention time (HRT) were 25 °C and 8 days, respectively. The low temperature and HRT were selected according to previous investigations in which these conditions were found appropriate for VFAs production [11,12]. Three scenarios were evaluated in terms of organic loading rate (OLR), namely: scenario 3B (48 days, OLR = 3 g COD/Ld before starvation), scenario 3A (38 days, OLR = 3 g COD/Ld after starvation (feeding re-start)) and scenario 9R (32 days, OLR = 9 g COD/Ld, recovery stage). The biological process was considered at steady state condition when VFAs resulted in a constant value and the reactor was operated during 3-HRTs. As samples were taken consecutively once the process had achieved the steady-state, multiple time point did not show variation. For this reason, samples collected and analyzed along the steady state offered a constant trend. pH was monitored but not controlled during the experiment. The starvation period lasted 14 days (2 weeks). The selection for this starvation period was based on the fact that this would be the time to recover an algal based system operating at hydraulic retention time of 4 days (typical value for urban wastewater treatment by means of algae consortium). In this manner, this study attempted to simulate a lack of feeding for 14 days due to a crash in the microalgae production system.

### 2.3. Analytical Methods

Total and soluble COD and NH_4_^+^ were measured twice per week using test kits (Merck, ISO 15705, ISO 000683, Darmstadt, Germany). Similarly, VFAs were measured by liquid chromatography (HPLC) an analyzed through an Agilent 1260 HPLC-RID (Agilent, Santa Clara, CA, USA) equipped with a Cation H Refill Cartridge Microguard column (Bio-Rad, Hercules, CA, USA) and an Aminex HPX-87H ion exclusion column (300 × 7.8 mm I.D.) (Bio-Rad). The biological process was considered at steady state condition when VFAs resulted in a constant value over three sampling points and the reactor was operated during three HRTs.

### 2.4. DNA Extraction

At the steady-state, samples (15 mL) were collected and frozen at −20 °C. The kit “FastDNA SPIN Kit for Soil” (MP Biomedicals, LCC, Illkrich, France) was used to extract DNA according to the protocol provided by the manufacturer. A nanodrop (LVis Plate, BMG LABTECH) was used to quantify the amount of extracted DNA (ng/mL) and analyze its quality by measuring 260/280 and 260/230 ratios. The primers used for the amplification of the 16S rRNA gene were 341F and 805R (F – CCTACGGGNGGCWGCAG and R – GACTACHVGGGTATCTAATCC), which targeted the hypervariable regions V3 and V4 of both bacteria and archaea [13]. Amplicons resulting from PCR were sequenced on a MiSeq Sequencer (Illumina, San Diego, CA, USA) and by Life Sequencing (University of Valencia, Spain) with MiSeq reagent kit v3 (600-cycle). 50 ng were amplified following the 16S Metagenomic Sequencing Library Illumina 15044223 B protocol (Illumina). In brief, the first amplification step, primers were designed containing: 1) a universal linker sequence allowing amplicons for incorporation indexes and sequencing primers by Nextera XT Index kit (Illumina); and 16S rRNA gene universal primers [13] and in the second and last amplification indexes were included. Libraries were quantified by fluorimetry using Quant-iT™ PicoGreen™ dsDNA Assay Kit (Thermofisher, Waltham, MA, USA) and pooled prior to sequencing on the MiSeq platform (Illumina), configuration 300 cycles paired reads. The size and quantity of the pool were assessed on the Bioanalyzer 2100 (Agilent) and with the Library Quantification Kit for Illumina (Kapa Biosciences), respectively. PhiX Control library (v3) (Illumina) was combined with the amplicon library (expected at 20%). Sequencing data were available within approximately 56 h. Image analysis, base calling and data quality assessment were performed on the MiSeq instrument. The resulting sequences were split taking into account the barcode introduced during the PCR reaction, while R1 and R2 reads were overlapped using PEAR program version 0.9.1 [14] providing a single FASTQ file for each of the samples. Quality control of the sequences was performed in different steps, (i) quality filtering (with a minimum threshold of Q20) was performed using fastx tool kit version 0.013, (ii) primer (16s rRNA primers) trimming and length selection (reads over 300 nts) was done with cutadapt version 1.4.1 [15]. These FASTQ files were converted to FASTA files and UCHIME program version 7.0.1001 was used in order to remove chimeras that could arise during the amplification and sequencing step. Those clean FASTA files were BLAST [16] against NCBI 16s rRNA database using blastn version 2.2.29+. The resulting XML files were processed using a python script developed by Lifesequencing S.L.-ADM (Paterna, Valencia, Spain) in order to annotate each sequence at different phylogenetic levels.

### 2.5. Statistical Analysis

Data were presented as mean values ± standard deviation of the mean and statistical significances was assessed by analysis of variance (ANOVA). Values were considered statistically significant when p value was lower than 0.05.

## 3. Results and Discussion

### 3.1. Effect of Starvation on Reactor Performance 

#### 3.1.1. Reactors Performance: VFAs Yields and Profiles

The negligible COD removal attained in scenario 3B (±5%, Table 1) indicated that operational conditions imposed were suitable for VFAs production. Low COD removals were the result of a poor methanogenesis and VFAs remained unconsumed. Most probably the use of an acidogenic inoculum with scarce methanogenic activity together with the low HRT imposed (8 days) contributed to VFAs accumulation by inhibiting the methanogenic stage. It should be highlighted that decreasing the HRT is considered a strategy to wash out methanogenic archaea as their growth rates are lower than those exhibited by acidogenic bacteria [17,18]. VFAs conversion yields attained at steady state (0.39 ± 0.03 COD-VFAs/COD in) were in agreement with previously reported values from microalgae biomass [11]. The measured soluble COD was about 16.41 ± 0.26 g/L. By taking into consideration the VFAs contribution in terms of COD (11.82 ± 0.96 g/L), it could be stated that the acidification stage (acidogenesis and acetogenesis) was efficient since the COD-VFA/sol COD ratio was 0.72.

**Figure 1:**
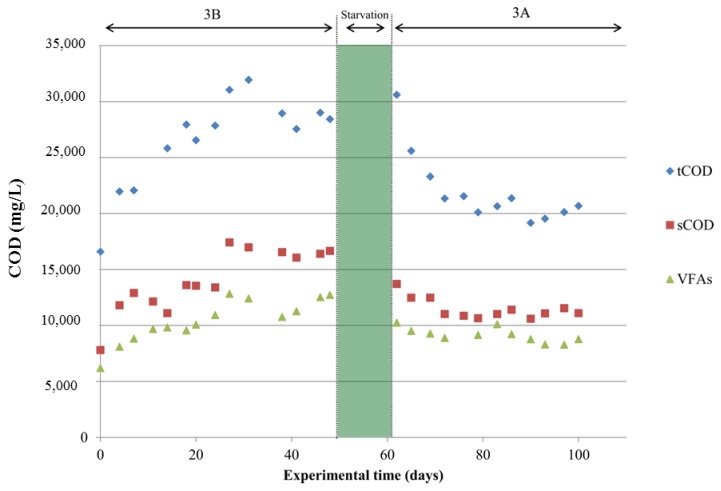
Main operational parameters assessed during reactor operation: total/soluble chemical oxygen demand (tCOD and sCOD) and volatile fatty acids (VFAs).

After stable operation, the system was subjected to a starvation period of two weeks. Starvation length is quite arbitrary in scientific literature. While some studies employ long-term starvation [19], others evidenced modest changes with just one day of starvation [20]. The effect of the lack of feeding can affect microbial activities [21], impacting ultimately the bioprocess efficiency. In the present investigation, after the starvation period, the reactor was operated at the same initial conditions. However, results in terms of COD elimination were quite different. In scenario 3A, COD removal increased up to approximately 33% (Table 1), and thus the organic matter conversion yield into VFAs decreased to 0.30 ± 0.02 COD-VFAs/CODin. With regard to the acidification stage, similarly to scenario 3B, high values were recorded when taking into consideration the VFAs contribution to the soluble COD (0.8 COD-VFA/sol COD ratio). This feature evidenced that the formation of VFAs was not affected by the starvation period. Lastly, when comparing effluent sol COD/tot CODin of both scenarios, a similar ratio was observed (0.58 ± 0.1 in 3B and 0.55 ± 0.1 in 3A). Therefore, it could be stated that starvation did not affect the fermentative stages (hydrolytic and acidogenic), but it had an influence in the methanogenic stage, as COD removal increased. The starvation period most probably contributed to the development of the archaea community because no effluent was extracted from the acidogenic reactor in 14 days. Additionally, archaea species might have recovered due to the lower NH_4_^+^ concentration detected after starvation. The trade-off of the main parameters evaluated is shown in Figure 1.

**Figure 2:**
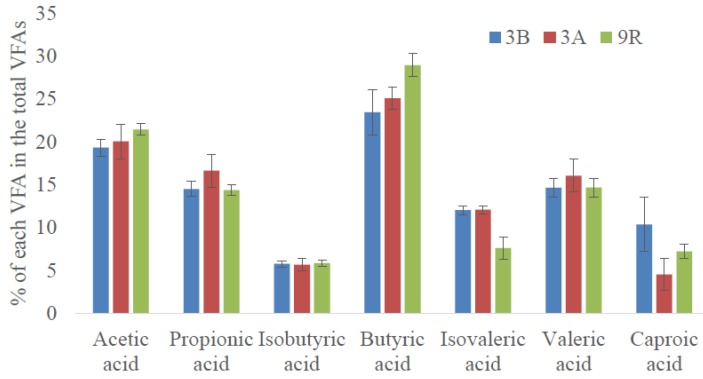
VFAs profiles exhibited at the stationary state of the different scenarios (3A, 3B, and 9R).

In terms of VFAs profile distribution, butyric acid was the VFA exhibiting the highest percentage (23% ± 2% of total VFA as COD), followed by acetic acid (20% ± 1% of total VFA as COD) and the odd chain VFAs (15% ± 1% of total VFA as COD of propionic and valeric acids). Generally, acetic, propionic, and butyric acids are the main products when microalgae biomass is subjected to AD [22,23]. As seen in Figure 2, this trend was maintained after starvation (scenario 3A). The only remarkable difference was attained for caproic acid that decreased from 10% ± 3% to 4% ± 2% of total VFA as COD (*p* < 0.05). However, the differences before and after starvation were minimal and thus, it can be pointed out that implemented disturbances did not greatly affect VFAs distribution.

#### 3.1.2. Microbial Population Dynamics 

**Figure 3:**
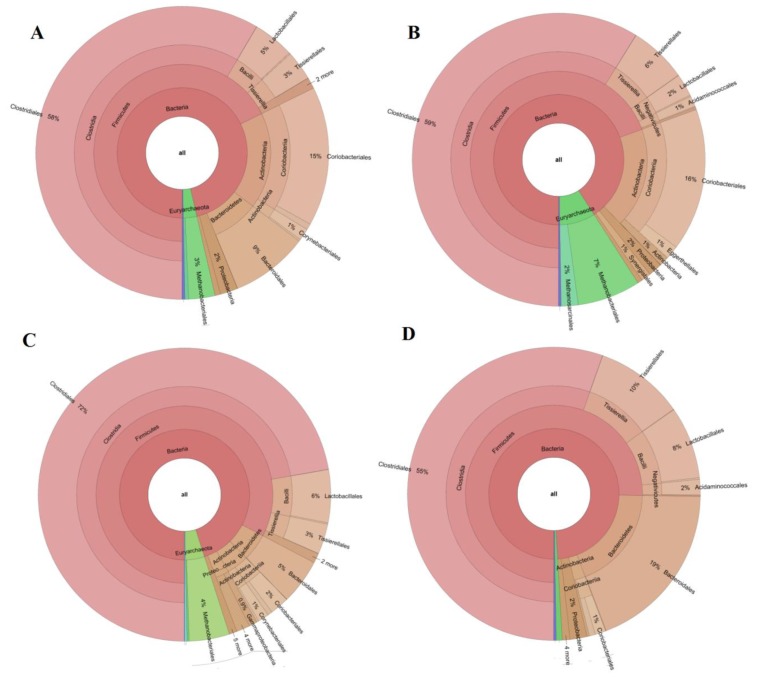
Krona graphics extracted from each scenario: 3B(**A**); starvation (**B**); 3A(**C**); 9R(**D**).

In order to link fermenters chemical outputs with the existing microbiome, microbial populations were analyzed before and after the starvation period (scenarios 3B and 3A, Figure 3). Anaerobic populations were evaluated in terms of relative abundance (%). The bacterial community before starvation consisted of Firmicutes (68%) as the major phylum, followed by Bacteroidetes (10%), and Actinobacteria (18%). Samples taken immediately after starvation, before restarting the feeding, showed no differences in terms of Firmicutes and Actinobacteria, while in the case of Bacteroidetes, the population drastically decreased to 0.5%. In addition, even though the relative abundance of Firmicutes was not affected, a decrease in Bacilli and an increase in Tissierellia class were observed (Figure 3A,B). Reactor operation after the starvation period returned Tissierellia and Bacilli values to those showed initially and gave rise to a sensitive increase in Firmicutes (Figure 3C). This might be attributed to Clostridia class, which increased from 58% to 72% (before and after starvation) with respect to the total sequences analyzed (Figure 3A–C). 

In general terms, the predominance of Firmicutes agreed with previous research studies dealing with the production of VFAs from microalgae biomass [11]. In fact, the bacterial population is markedly different to the obtained herein when the digestion is targeted for biogas production. Bacterial community when biogas is the end-product is mainly represented by Cloroflexi (under low ammonium levels [24]) or by Proteobacteria [25] while the relative abundance of Firmicutes is considerably lower [25,26]. Other studies stated that a high presence of Firmicutes is related to poor biogas production performance, which is in fact the scenario sought herein [7]. Ammonium and ammonia might be toxic for anaerobes and therefore it should be carefully analyzed. With regard to ammonium, the concentration determined during scenario 3B was 1.28 ± 0.02 g NH_4_^+^/L (Table 1). In this particular case, ammonium concentration was high but not yet in the inhibitory concentration range considered for un-acclimated inoculum (1.7–1.8 g/L, [27]). 

More importantly, the percentage of Euryarchaeota community (archaea) displayed a significant increase during starvation confirming the recovery of this community (Figure 3). Note worth to mention that the main strain determined among this population was *Methanobacterium*. This strain has been claimed to be a hydrogenotrophic methanogen [28]. In this context, there are two major methanogenic pathways: a) the acetoclastic pathway and b) the hydrogenotrophic pathway. Additionally, syntrophic acetate oxidizing bacteria (SAOB) might occur. These species oxidize acetate and produce H_2_ and CO_2_ or formate. This H_2_ generated might be used as well for hydrogenotrophic methanogenesis. 

Acetoclastic pathway is mediated by families related with Methanosarcinaceae spp. and Methanosaetaceae spp., while species belonging to order Methanomicrobiales spp., Methanobacteriales spp. (such as *Methanobacterium*), and Methanococcales spp., are responsible for the hydrogenotrophic pathway [29]. It should be highlighted that this latter methanogenic route prevails over the acetoclastic pathway when difficult methanogenesis environments are imposed. As a matter of fact, the acetoclastic archaea are more sensitive than hydrogenotrophic species [30]. For instance, digesters operating at high ammonium or VFAs concentrations, which can be potentially toxic, have shown hydrogenotrophic pathway preference for methanogenesis [4,31]. These adverse conditions for methanogenesis were also evidenced in scenario 3B while immediately after the starvation period, methanogens activity resumed as it could be seen by archaea population increase after starvation in Figure 3. This feature is in agreement with Kim et al., (2015) who pointed out that under starvation conditions methanogens are able to enter a quiescent state until favorable conditions for growth are attained again. The lower conversion yield in terms of COD-VFAs/CODin attributed to the consumption of VFAs was also related to the presence of syntrophic acetate oxidizing bacteria (SAOB). SAOB are normally working together with their hydrogenotrophic counter partners to keep an optimum hydrogen trade off in the anaerobic system. Acetate oxidation only proceeds when the hydrogen level is kept low by hydrogenotrophic methanogens consumption [32]. Whereas, the presence of Chloroflexi has been negatively correlated with VFAs production, other phylum such as Firmicutes prevails in environments devoted for VFAs production [11,33]. SAOB are affiliated with Firmicutes phylum, more particularly to Clostridia class (*Thermacetogenium phaeum, Tepidanaerobacter acetatoxydans,* or *Syntrophaceticus schinkii*), Tissierellia class (*Clostridium ultunense*) and Thermotogae phylum (*Pseudothermotoga lettingae*) [34,35]. However, other members of Firmicutes have been attributed to perform SAO activities. In fact, species belonging to Clostridia class have been previously related with the SAO pathway [36]. In this sense, the highest COD removals and lowest COD-VFAs/CODin conversions were attained under scenario 3A, which showed the highest Clostridia population (72%). Moreover, the methanogens recovery during starvation might also be linked to the lower ammonium concentration of the digestates after starvation (0.89 ± 0.02 g NH_4_^+^/L, Table 1). Indeed, the nitrogen mineralization percentage was not recovered since ammonium levels in the effluents after starvation did not reach the same concentration as in scenario 3B.This could be explained by the different fate of carbon and nitrogen during AD [37]. In this case, it seems likely that nitrogen mineralization did not recover its initial efficiency.

### 3.2. Recovery Strategy: OLR Increase

#### 3.2.1. Reactors Performance VFAs Yields and Profiles during Fermenter Recovery 

The OLR has been reported as a bioengineering management tool to shape anaerobic digesters performance [7]. Aiming at recovering the organic matter conversion into VFAs, OLR was stepwise increased to reach 9 g COD/Ld (scenario 9R).This strategy resulted in the same conversion yield (0.39 ± 0.04 COD-VFAs/CODin) attained before starvation (3B). Likewise, COD removal decreased to values similar to scenario 3B (Table 1). Moreover, it should be highlighted that ammonium concentration in scenario 9R increased to 2.89 ± 0.23 g NH_4_^+^/L (Table 1) and VFAs concentration was 18.3 ± 0.3 g/L. Both parameters, ammonium and VFAs, were above the threshold limits identified for proper biogas production [27,31]. These two facts likely contributed to methanogenesis inhibition resulting in similar COD removals values and organic matter conversion into VFAs previously showed in 3B. The acidogenesis stage also remained stable when compared with the two previous scenarios (0.73 COD-VFA/sol COD ratio) indicating that acidogenesis was not affected by starvation, ammonium concentration or the OLR increase. With regard to the hydrolysis stage, the increase in OLR (scenario 9R) also supported an increase in the ratio effluent sol COD/tot CODin (0.59), which was similar to the values attained in scenario 3B. Based on the effluent ammonium concentration attained during scenario 9R, it could be stated that nitrogen mineralization efficiency was similar to scenario 3A. In this manner, the diminished activity in nitrogen mineralization registered after the starvation still remained after the OLR increase. Thus, organic matter conversion yield into VFAs was recovered but not the nitrogen mineralization. It should be highlighted that the goal of this recovery strategy was to obtain the same VFA conversion yield as before of the starvation period.

In terms of VFA distribution, slight differences in VFAs content were determined even though a similar profile trend to the one obtained in scenario 3B was observed. In general terms, butyric (29% ± 1% of total VFA as COD) and acetic acids (21% ± 1% of total VFA as COD) were the dominant VFAs. As it was aforementioned, this is a normal trend in microalgae biomass AD. These values represented slightly higher values than the ones obtained in scenario 3B. At the expenses of the increased percentage of those two acids, lower percentages of the longest VFAs (C5 and C6) were attained (Figure 2).

#### 3.2.2. Microbial Population Dynamics during Fermenter Recovery

**Figure 4:**
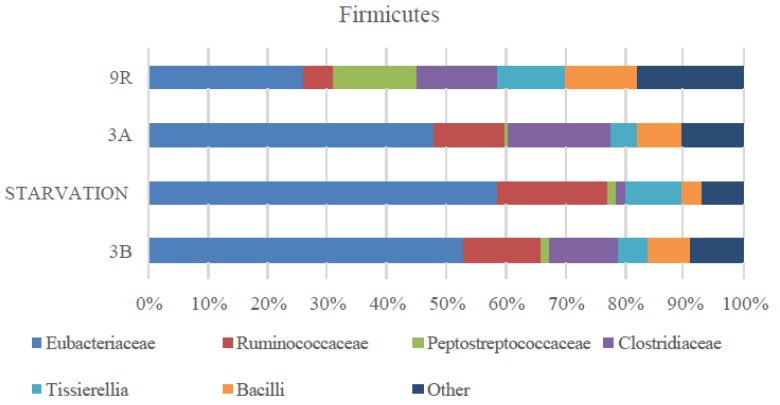
Firmicutes phylum distribution at the different scenarios assessed (3B, starvation, 3A, and 9R).

As seen in Figure 3, microbial population changed when increasing OLR to 9 g COD/Ld (scenario 9R). One of the main differences in scenario 9R was associated to the stepwise decrease of the Euryarchaeota population with respect to the starvation period and 3A. In this case, archaea accounted for 1% of the microbial population. This fact combined as aforementioned with higher VFAs productions and NH_4_^+^ concentration, which might entail toxicity for the anaerobic populations, weakened the organic matter removal in the system. This fact underpinned the low COD removal values determined in this scenario. When compared to scenario 3A, not only archaea community decreased but also a marked increase in Bacteroidetes phylum was observed (20%, Figure 3D). Opposite to that, the increase in OLR did not affect the Actinobacteria population percentage that stayed at low values (2%). In spite of the similar VFAs conversion values in scenarios 3B and 9R, their microbial populations were slightly different. More specially, Bacteroidetes and Euryarchaeota were present at similar percentages while Firmicutes increased to 75% and Actinobacteria decreased to 2% at 9R. Despite the increased percentage of Firmicutes, the prevalence of Clostridiales within this phylum attained its initial value (58% and 55% in scenarios 3B and 9R, respectively). Some other remarkable changes within the anaerobic microbiome at OLR 9 g COD/Ld included the drastic decrease of Ruminococcaceae and Eubacteriaceae and the increase of Peptrostreptococcaceae (Figure 4). Therefore, even though Firmicutes phylum remained similarly high to the previous scenarios, the relative abundance of the bacterial class was quite different. Overall, it could be concluded that despite of the conversion yield recovery in terms of VFAs production, microbial community did not return to the initial structure (scenario 3B) after recovery (scenario 9R). In this sense, the microbial systems developed under scenario 9R and 3B were functionally redundant indicating that the new microbial community could maintain similar performance efficiency supporting similar VFAs yields. This behavior was previously reported in literature when targeting biogas production [8,38]. However, to the best of the knowledge authors, AD robustness was not proven previously for VFAs production.

## 4. Conclusions

The starvation period affected the overall process performance (VFAs yields and nitrogen mineralization) as well as the microbiome involved. More specifically, methanogenic archaea were able to thrive after the lack of feeding resulting in an increase in COD removal via the hydrogenotrophic pathway. The recovery strategy of applying an OLR increase recovered conversion values showed initially (0.39 ± 0.04 COD-VFAs/CODin). This approach weakened methanogenesis and contributed to maintain archaea and Clostridia levels similar to those showed initially. Remarkably, microbial systems developed (represented by Firmicutes) were functionally redundant since the new community could maintain similar performance efficiency highlighting the robustness of anaerobic fermentation for VFAs production.

## Figures and Tables

**Table 1:** Effluent results of the different parameters assessed at the different scenarios.

Scenario	OLR (g COD/Ld)	%COD Removal	Soluble COD (g COD/L)	VFAs (g COD/L)	COD-VFAs/CODin	NH_4_^+^ (g/L)	pH
3B	3	5.1 ± 2.2	16.42 ± 0.26	11.82 ± 0.96	0.39 ± 0.03	1.28 ± 0.02	6.3 ± 0.1
3A	3	32.5 ± 2.7	11.12 ± 0.33	8.90 ± 0.69	0.30 ± 0.02	0.89 ± 0.02	6.1 ± 0.1
9R	9	3.3 ± 1.8	38.16 ± 0.32	27.92 ± 2.90	0.39 ± 0.04	2.83 ± 0.02	6.3 ± 0.1

OLR: Organic loading rate; %COD removal: chemical oxygen demand removal; soluble COD: soluble chemical oxygen demand; VFAs: Volatile fatty acids; COD-VFAs/CODin: Volatile fatty acids in terms of chemical oxygen demand out of the total chemical oxygen demand fed in the system.

## Data accessibility

These sequence data have been submitted to GenBank database in a project (accession number PRJNA529178).
